# Nicolau syndrome following intramuscular benzathine penicillin injection: a case report

**DOI:** 10.11604/pamj.2020.37.276.21850

**Published:** 2020-11-26

**Authors:** Walaza Phiri, Maen Shulammite Musonda, Kebby Kyakilika, Michelo Haluuma Miyoba, Malan Malumani

**Affiliations:** 1 Department of Pediatrics and Child Health, Livingstone Central hospital, Livingstone, Zambia,; 2 Department of Pharmacy, Livingstone Central Hospital, Livingstone, Zambia,; 3 Department of Surgery, Livingstone Central Hospital, Livingstone, Zambia,; 4 Mulungushi University, School of Medicine and Health Sciences, Department of Surgery, Mulungushi University, Livingstone, Zambia,; 5 Department of Internal Medicine, Dermatology and Venereology Unit, Livingstone Central Hospital, Livingstone, Zambia

**Keywords:** Livedo-like dermatitis, embolia cutis medicamentosa, Nicolau syndrome (NS), intramuscular injections, case report

## Abstract

Nicolau syndrome (NS) is a rare injection site reaction, following intramuscular injection of drugs characterized by severe pain, skin discoloration and varying level of tissue necrosis. The case outcomes vary from severe pain, atrophic ulcers to sepsis and limb amputation. We describe a case of the five-year-old girl with diagnosis of NS after intramuscular benzathine penicillin injection. The case was complicated with above the knee amputation of lower limb. This case report intends to remind clinicians that such rare cases should always be thought of in all patients receiving whatsoever drug via intramuscular injections.

## Introduction

Nicolau Syndrome (NS) is a rare livedo-like dermatitis which is a complication caused by intramuscular injection of various medication [1]. It is also known as embolia cutis medicamentosa [2]. Several drugs such as penicillin, non-steroidal anti-inflammatory drugs [3], corticosteroids [4] and local anesthetics have been implicated as the cause of NS. Nicolau syndrome has also been reported after intravenous, subcutaneous, or intraarticular injection [4,5]. Nicolau syndrome was first described in patients who were receiving intramuscular injection of bismuth salt in the treatment of syphilis in the early nineteenth century [6]. The pathogenesis of NS is unknown but sympathetic nerve stimulation, prostaglandin synthesis block, embolic occlusion, inflammation and physical obstruction of blood vessels have been suggested. However, the development of the acute vasospasm following intravenous or around the vein injection is the mostly widely accepted hypothesis [7,8]. This syndrome presents with intense pain immediately or soon after the injection with associated a bluish discoloration livedo-like. In some clients they may present with a progressive hyperemia, redness and local ischemic necrosis of the skin and adipose tissue if no intervention is instituted [9]. We discuss a case of Nicolau syndrome in a 5-year-old Zambian girl after intramuscular administration of benzathine penicillin for acute on chronic tonsillitis.

## Patient and observation

**Figure 1:**
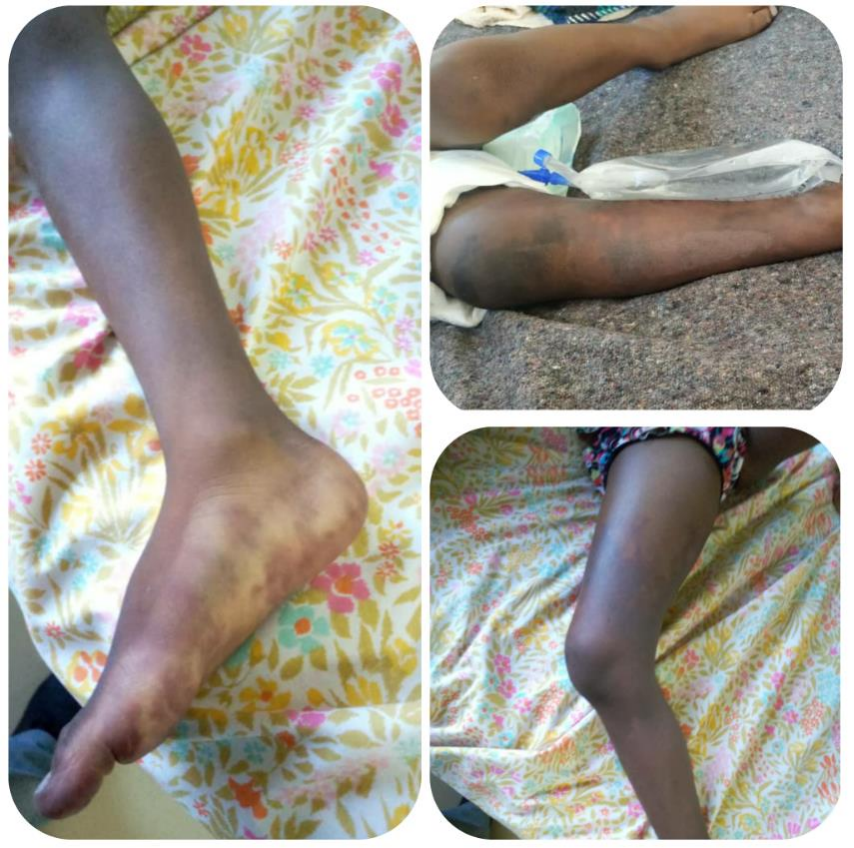
the right lower limb of the patient in a paralytic state showing well defined violaceous patchy regions of the limb from different angle

A five-year-old girl presented to the pediatric emergency room with acute onset of pain of the right lower limb, coldness of the limb and inability to use the limb. The patient was given benzathine penicillin intramuscular injection on the same limb for acute on chronic tonsillitis 8 hours prior to presentation. The right lower limb pain was associated with coldness of the limb and dark discoloration. The pain and discoloration were said to be getting worse with time. However, there was no history of fevers, trauma, sickle cell disease or bleeding tendencies. Examination revealed swollen right lower limb with dark discoloration from the toes to mid-thigh as in Figure 1. The leg was cold with weak popliteal and dorsalis pedis pulses compared to the contralateral limb. Sensation was intact and power was zero. Systemic examination was unremarkable. A diagnosis of compartment syndrome of the right thigh secondary to Nicolau syndrome was made. Investigations done were full blood count (FBC), urea, electrolytes, creatinine, liver enzymes, INR and Doppler ultrasound of the right lower limb. FBC results were normal. Urea and creatinine were elevated 13.5mmol/l and 242.2umol/l respectively. Alanine aminotransferase (ALT) and aspartate aminotransferase (AST) were 997.7U/L and 115.6IU/L. International normalization ratio (INR) was 1.57.

**Figure 2:**
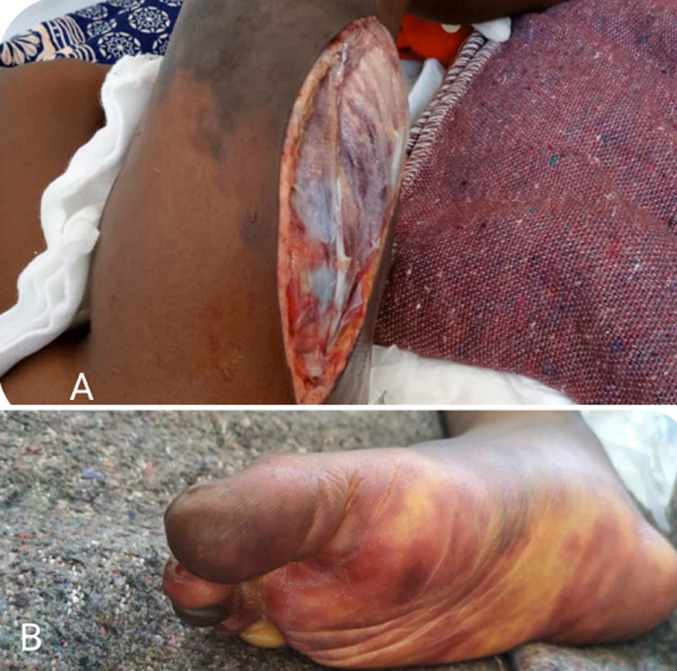
A) showing muscle necrosis at the fasciotomy site with some areas still showing signs of viability-bleeding; B) cyanotic-violaceous discoloration of the right foot

**Figure 3:**
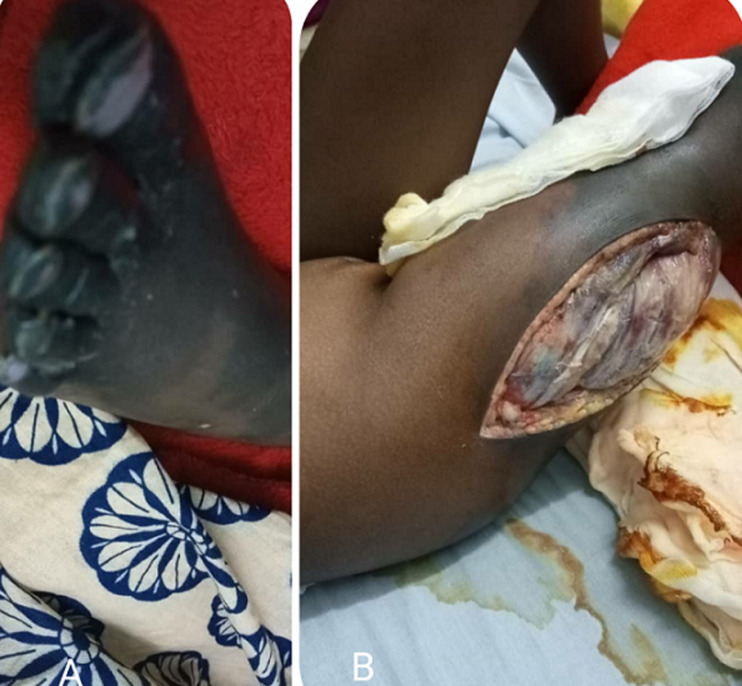
A) worsening dry gangrene and mummification of the toes and foot; B) worsening of muscle necrosis with bacterial super infection of the surgical wound

**Figure 4:**
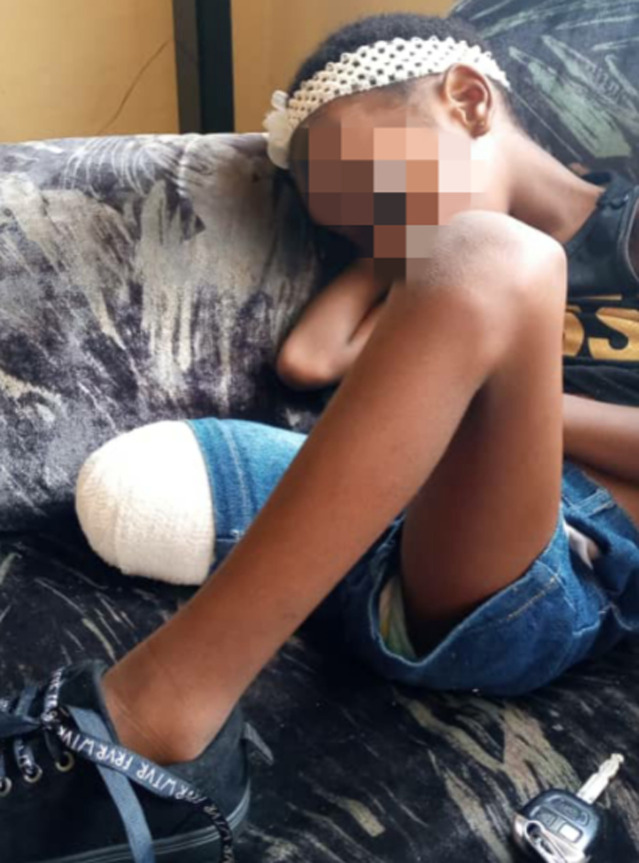
our patient resting at home after an occupation rehabilitation session at the physiotherapy department

An emergency fasciotomy was done 24hrs after presentation after surgeons reviewed the patient and intra-op findings were osteofascial compartment bulging on first incision with free clear fluid and minimal bleeding as shown in Figure 2. Patient was then started on broad spectrum antibiotics; imipenem 250mg thrice a day (TDS) IV, metronidazole 200mg IV, analgesia and tramadol 25mg twice a day intramuscular (B.D IM). The acute kidney injury (AKI) was managed conservatively by restricting fluid intake and avoiding nephrotoxic drugs. AKI resolve after 5 days with urea and creatinine dropping to 3.36mmol/l and 37.26umol/l. ALT and AST also dropped to 64.1U/L and 180.3IU/l with albumin of 18.5g/l. However, there was no improvement in the perfusion of the right leg and foot two days after fasciotomy. A diagnosis of dry gangrene was made and patient was planned for a below knee amputation but parents denied consent. The parents continued being counselled on the need for an amputation. After 4 days post fasciotomy, patient started having spiking temperatures despite being on imipenem. The swelling worsened with intense pain in the right thigh and right thigh skin started developing blisters. A diagnosis of dry gangrene with superimposed wet gangrene was made 13 days after fasciotomy as indicated in Figure 3. Parents were counselled for an above knee amputation but consent was denied again. Patient developed convulsions which were attributed to raised body temperatures. The seizures were controlled by diazepam 7mg intravenous (I.V) stat. Four days later, the parents accepted for an above knee amputation (AKA) to be done. The AKA was done and intra-op findings were cooked muscles of the right thigh due to poor perfusion. Patient was then put on ceftriaxone, metronidazole and tramadol. The temperatures settled after the AKA. Wound was then exposed after 24hrs and daily cleaning was ordered. The patient was later discharge and she is currently undergoing occupational therapy sessions at the physiotherapy department as in Figure 4.

## Discussion

Nicolau syndrome mostly present as severe pain around the injection site, followed by erythema, livedo reticularis or hemorrhagic patches immediately after injection [10]. The livedo discoloration is usually with sharp, angulated margins and sometimes it assumes a reticulate pattern which has been referred to as noninflammatory retiform purpura, livedo-like dermatitis and livedoid dermatitis with severe necrosis. Apart from the discoloration, involvement of the deeper subcutaneous tissues including necrosis and ulceration of the skin, muscle tissue, and subcutaneous adipose may also occur [11]. However, some patients may present with paralysis of the limb involved which has been attributed to the force of injection from the gluteal vessels into the internal iliac arteries and ischemia of the sciatic nerve [12]. This was a similar prestation of our patient. She experienced the pain just after the injection as an out-patient. Thus, the mother thought it was the usual normal injection pain that all patients who receive intramuscular injections feel. Eight hours after the injection, the mother noticed the child was unable to move the limb and it had changed color. The delay in presentation to the hospital and in making the diagnosis of Nicolau syndrome in our patient compounded the progression of the condition. The diagnosis was only made 24 hours after presentation to the hospital by then the limb was in a paralytic phase and the peripheral pulses non-palpable. And they considered compartment syndrome and she was rushed in to operation room for fasciotomy. This revealed or showed a huge knowledge gap among our clinicians. Senel Engin, 2012, in his article describes that early incitation of treatment with anti-coagulant may reverse tissue damage in NS [10].

The diagnosis of NS is mainly clinical. Tissue diagnosis based on skin biopsy usually shows necrosis of dermis and subcutaneous tissue and muscle biopsy shows focal vascular thrombosis and inflammatory infiltrate in acute phase [12]. Doppler ultrasound is usually normal in patients with NS [10] as was in the case in our patient. Other differential diagnosis to be considered in patient with NS include cutaneous cholesterol embolia (common in adults), vasculitis, and cutaneous embolization of cardiac myxoma. Management of NS should be multidisciplinary involving clinicians, pharmacists, nurses and physiotherapy. There is no consensus on the treatment of NS so far however, early institution of treatment has been shown to reduce the tissue necrosis [11]. In the initial phase, because of the severe intense pain, conservative pain control and dressing is advised [8]. In the acute phase, treatment is aimed at improving vascularity in the affected limb, hence the use of vasoactive agents such as subcutaneous heparin and oral pentoxifylline [10]. In our case no heparin was administrated. Topical or intralesional steroids have been used to reduce inflammation [8]. Surgical debridement of ulcers is also important as it reduces infection and enhances wound healing [13]. However, failure to recognize the extent of fat necrosis and poor blood supply leads to inadequate debridement and poor wound healing which can predisposes the patient to repeated cycles of infection leading to extensive scarring, soft tissue indentation, skin graft and sometimes amputation [12]. Empirical antibiotics also play a role in management of NS in cases of infection complications. Pharmacist have to ensure that the drugs being given are checked for expiry dates and dispensed to the correct patient with the correct diagnosis and make sure that the patient understands the side effects, adverse effects of the drugs.

Adil M *et al*. 2017 alludes that Nicolau syndrome is an avoidable complication. It is also important for nurse to follow the correct method of intramuscular injection by using the “Z track method”. Injection should be in the upper quadrant of the buttock and aspirating the needle before injecting the medication to ensure that the drug is not administered into the vessels [14]. Patients should be explained to on the adverse effects of intramuscular drug injection and the authors recommend that clinicians should be highly suspicious of this rare condition in any patient who has received an intramuscular drug injection for whatever condition as long as they complain of severe stubbing pain on the injection site.

## Conclusion

Nicolau syndrome is a rare complication of intramuscular drug injection. Clinicians should have a high index of suspicion of NS if patient presents with severe pain following intramuscular injection. Treatment with anti-coagulants should be quick if there are no absolute contraindications and involves a multi-disciplinary approach.

## References

[1] Ozlu E, Baykan A, Ertas R, Ulas Y, Ozyurt K, Avci A (2017). Case report: Nicolau syndrome due to etofenamate injection. F1000Res.

[2] Agarwal A, Kabra A, Jain R, Bhargava G (2018). Nicolau´s Syndrome (embolia cutis medicamentosa). J Assoc Physicians India.

[3] Nayci S, Gurel MS (2013). Nicolau syndrome following intramuscular diclofenac injection. Indian Dermatol Online J.

[4] Cherasse A, Kahn M-F, Mistrih R, Maillard H, Strauss J, Tavernier C (2003). Nicolau´s syndrome after local glucocorticoid injection. Joint Bone Spine.

[5] Sonntag M, Hodzic-Avdagic N, Bruch-Gerharz D, Neumann NJ (2005). Embolia cutis medicamentosa after subcutaneous injection of pegylated interferon-alpha. Hautarzt.

[6] Corazza M, Capozzi O, Virgilit A (2001). Five cases of livedo-like dermatitis (Nicolau´s syndrome) due to bismuth salts and various other non-steroidal anti-inflammatory drugs. J Eur Acad Dermatol Venereol.

[7] Luton K, Garcia C, Poletti E, Koester G (2006). Nicolau Syndrome: three cases and review. International Journal of Dermatology.

[8] Kim KK, Chae DS (2015). Nicolau syndrome: A literature review. World Journal of Dermatology.

[9] Ozcan A, Senol M, Aydin EN, Aki T (2005). Embolia cutis medicamentosa (Nicolau Syndrome). Clin Drug Investig.

[10] Senel E (2012). Nicolau syndrome as an avoidable complication. J Family Community Med.

[11] Nischal K, Basavaraj H, Swaroop M, Agrawal D, Sathyanarayana B, Umashankar N (2009). Nicolau Syndrome: An Iatrogenic cutaneous necrosis. J Cutan Aesthet Surg.

[12] Sousa RD, Dang A, Rataboli PV (2008). Nicolau syndrome following intramuscular benzathine penicillin. Journal of Postgraduate Medicine.

[13] Kilic I, Kaya F, Ozdemir AT, Demirel T, Celik I (2014). Nicolau syndrome due to diclofenac sodium (Voltaren®) injection: a case report. Journal of Medical Case Reports.

[14] Maneshi A, Ravi S, Salehi MR, Hasannezhad M, Khalili H (2017). Nicolau Syndrome. Arch Iran Med.

